# A comparison of brown fat tissue related hormone levels in metabolically healthy and unhealthy individuals with obesity

**DOI:** 10.1007/s12020-024-03960-8

**Published:** 2024-07-15

**Authors:** Hacer Hicran Mutlu, Saniye Koç Ada, Mehmet Uzunlulu, Hasan Hüseyin Mutlu, Mehmet Sargın, Aytekin Oğuz

**Affiliations:** 1 Department of Family Medicine, Faculty of Medicine, Istanbul Medeniyet University, Istanbul, Turkey; 2 Department of Biochemistry, Faculty of Medicine, Istanbul Medeniyet University, Istanbul, Turkey; 3 Department of Internal Medicine, Faculty of Medicine, Istanbul Medeniyet University, Istanbul, Turkey; 4 Department of Family Medicine, Faculty of Medicine, Health Sciences University, Istanbul, Turkey

**Keywords:** Obesity, Metabolism, Metabolic syndrome, White adipose tissue, Brown adipose tissue, Browning agents

## Abstract

**Purpose:**

One of the key functions of brown adipose tissue is its positive impact on metabolism. This study aimed to examine the potential involvement of brown fat-related hormones in the development of metabolically healthy obesity. Specifically, we sought to compare the levels of NRG4, FGF21, and irisin between metabolically healthy and unhealthy individuals with obesity.

**Methods:**

Patients with BMI ≥ 30 kg/m^2^ and aged between 20 and 50 years were included in the study. Among these patients, those who did not have any metabolic syndrome criteria except for increased waist circumference were defined as metabolically healthy obese. Age, gender, BMI, body fat, and muscle mass, matched metabolically healthy and unhealthy obese groups were compared in terms of FGF21, irisin, and NRG4 levels.

**Results:**

Metabolically healthy and unhealthy obese groups were similar in terms of age and gender. There was no difference between the two groups in terms of BMI, weight, total body fat, muscle, fat-free mass, distribution of body fat and muscle mass. No statistically significant difference was found between irisin, NRG4, and FGF21 levels between metabolically healthy and unhealthy individuals with obesity. It was found that irisin had a significant inverse correlation with BMI and body fat percentage.

**Conclusion:**

The present study showed no difference between metabolically healthy and unhealthy obese individuals in terms of irisin, FGF21, and NRG4 levels. The weak association between irisin and BMI and body fat percentage may suggest a potential link between irisin with metabolic health.

## Introduction

In recent years, obesity has become an epidemic in both children and adults and, obesity-related diseases such as type 2 diabetes, cancer, and cardiovascular disease have also increased [1].

Studies have shown that some individuals with obesity may not be exposed to the adverse events caused by obesity and may remain metabolically healthy. Despite being obese, their glucose, lipid, and blood pressure levels are in normal range [2]. Therefore, searching for etiopathogenesis of obesity and metabolic health and developing treatments for obesity have become a matter of curiosity. There are many articles in the literature on the relationship between brown adipose tissue and metabolic health [3–6], in the light of these statements we supposed that brown adipose tissue may be one of the factors that lead to these metabolic differences between metabolically healthy and unhealthy individuals with obesity.

Recently, brown adipose tissue has become intriguing, and various studies have been carried out on its function. One of the most important functions of brown adipose tissue is the favorable effect of this tissue on metabolic functions [7]. It exerts its favorable effects on metabolism in various ways. Firstly, it causes calorie burning and thus weight loss by increasing heat production. Secondly, it provides positive effects on glucose metabolism by increasing insulin sensitivity and glucose uptake and it has positive effects on lipid metabolism by decreasing hepatic lipogenesis and increasing fat oxidation [8]. If these favorable effects occur only with the existence of brown adipose tissue, it was thought that brown adipose tissue transplantation could also have a positive effect on metabolism and could be a therapeutic target, and therefore brown adipose tissue transplantation studies were performed in mice [9, 10]. In most of these studies, while favorable effects on glucose, lipid metabolism, and weight were observed in animals transplanted with brown adipose tissue [11–14], it was observed that transplanted brown adipose tissue cells were larger than endogenous brown adipose tissue cells and had less thermogenic gene expression [4, 9]. It was noticed that while transplanted brown adipose tissue lost its thermogenic function, it activated the thermogenesis of endogenous brown adipose tissue. [12]. Stanford et al. observed that when brown adipose tissue was transplanted into the visceral cavity of adult diabetic mice, interleukin 6 (IL-6) levels increased first, followed by an increase in fibroblast growth factor 21 (FGF21) levels and improved glucose tolerance [15]. Therefore, it is possible that the beneficial effects of brown adipose tissue are not due to the brown adipose tissue itself, but to the secretion of FGF21, IL-6, growth differentiation factor faktör 15 (GDF15), neuregulin 4 (NRG4), bone morphogenic protein 8b (Bmp8b), 12,13-dihydroxy-9Z-octadecenoic acid (12,13-diHOME), 9-hydroxy eicosapentaenoic asit (9-HEPE) and batokines such as follistatin [4, 8, 9, 16]. The browning effects of these batokines on white adipose tissue have a positive effect on metabolic health [3, 17].

In the light of these statements, we aimed to investigate whether brown fat-related hormones which have browning effect on white adipose tissue may be involved in the ethiopathogenesis of metabolic healthy obesity. The purpose of this study was to evaluate the difference between the levels of NRG4, FGF21, and irisin secreted from brown adipose tissue between metabolically healthy and unhealthy individuals with obesity with equal amounts and distribution of adipose tissue.

## Materials and methods

### Study population

Patients who consecutively applied to the obesity outpatient clinic of Göztepe Prof. Dr. Süleyman Yalçın City Hospital between 18.5.2021 and 01.04.2022, met the inclusion criteria, gave written consent that they agreed to participate were included in the study. Patients with BMI ≥ 30 kg/m^2^ and aged between 20 and 50 years were included in the study. Patients receiving antidiabetic, antihypertensive, and lipid-lowering treatment, steroid users, smokers, and/or alcohol users, pregnant women, patients with hypo/hyperthyroidism, malignant tumors, liver and kidney dysfunction, patients with secondary obesity, and cardiovascular disease, and patients who performed lifestyle changes in the last 3 months were excluded from the study.

Among these patients, those who did not have any metabolic syndrome criteria except for increased waist circumference (since increased waist circumference is detected in 85–95% of the persons with obesity) (blood pressure ≥130/85 mmHg, fasting blood glucose ≥100 mg/dl, triglycerides ≥150 mg/dl, HDL < 40 mg/dl in men, <50 mg/dl in women or those who use medication to reduce these parameters) were defined as metabolically healthy and those with any of these criteria were defined as metabolically unhealthy. The metabolically healthy definition of Van Vliet-Ostaptchouk et al. [18] was referred to in this study.

To determine the necessary sample size for this study, we aimed to detect a standardized effect size (Cohen’s d) of 0.5. Using G*Power© software, with a significance level (α) of 0.05 and power (1-β) of 0.80, we calculated the minimum sample size for each group as 51. In consideration of potential participant dropout, we adjusted our target sample size upward, aiming to recruit 126 participants in total which consisted of 61 metabolically healthy and 65 metabolically unhealthy people with obesity which matched in terms of age, gender, BMI, body fat, and muscle mass.

The study was approved by the Ethical Committee of the Clinical Research of Göztepe Training and Research Hospital with number 2020/0577 on 30.09.2020. (Clinical Trial Gov Number NCT05232695).

### Data collection tools

#### Sociodemographic characteristics

Participants’ age, gender, presence of childhood obesity, physical activity status, number of daily meals, inter-meal and nocturnal eating habits, presence of obesity in parents, siblings, and other family members, and co-morbidities were asked face to face by researchers.

#### Anthropometric measurements

The body fat, muscle, and fluid mass were measured by bioelectrical impedance method by entering participants’ age, gender, and height data (TANITA MC 780-MA, Tokyo, Japan).

The waist circumference of the patients was measured by the same person with a tape measure at the narrowest point between the anterior superior iliac processes and the lower edge of the costae, and hip circumferences was measured at the widest point of the hip in a slightly expired state.

#### Blood pressure measurements

The blood pressure of the participants was measured with a sphygmomanometer.

#### Laboratory measurements

Fasting blood glucose, insulin, c-peptide, HbA1c, lipid parameters, vitamin B12 and D, urea, creatinine, uric acid, and TSH levels which are routinely tested in the obesity outpatient clinic were recorded from their patient files. A 10 ml blood sample was collected from the patients by standard phlebotomy in vacutainer tubes with EDTA. Blood samples were centrifuged at 3500 rpm for 5 min and stored at −80 °C until the day of study. Then, FGF21, irisin, and NRG4 measurements in serum samples were performed using a commercial ELISA kit according to the company protocol.

#### Data analysis

SPSS (ver.25) statistical package program was used for data analysis. Descriptive statistical methods (mean, standard deviation, median, frequency, ratio, minimum, maximum) were used to evaluate data. Normally distributed data were shown as mean ± standard deviation and the data which was not distributed data were shown as median (minimum-maximum). Categorical data was presented as “percentage”. In addition to descriptive data, for the comparison of two groups with normal distribution Student’s t-test was used and for the comparison of two groups with non-normal distribution Mann–Whitney U test was used. The relationships between quantitive variables were evaluated by Pearson and Spearman correlation tests. *P* < 0.005 was accepted as statistically significant.

## Results

Sociodemographics of participants are shown in Table 1. The mean age of the individuals with obesity was 34.8 ± 9.04. 82.5% of the participants were female, 31% of them had a history of childhood obesity and 68.3% of them had a family history of obesity. Most of the patients with obesity had low levels of physical activity (75.4%) (Table 1).

**Table 1:** The comparison of sociodemographic characteristics of individuals with obesity according to their metabolic phenotypes

		Metabolic phenotype			
		Metabolically unhealthy	Metabolically healthy	Total sample	
		% (n) Mean±SD	% (n) Mean±SD	% (n) Mean±SD	*p*
Gender	Female	80 (52)	85.2 (52)	82.5 (104)	0.44
	Male	20 (13)	14.8 (9)	17.5 (22)	
Age		35.7 ± 8.2	33.9 ± 9.8	34.8 ± 9.04	0.39
The history of childhood obesity		27.7 (18)	34.4 (21)	31 (39)	0.41
Physical activity	<30 min/week	81.5 (53)	75.4 (95)	75.4 (95)	0.13
	3 times/week, at least 30 min	6.2 (4)	11 (14)	11 (14)	
	5 times/week, at least 30 min	12.3 (8)	13.5 (17)	13.5 (17)	
The number of daily meals		2.4 ± 0.5	2.3 ± 0.6	2.4 ± 5.8	0.43
Snack habit		55.6 (35)	64.4 (38)	59.8 (73)	0.32
Night-time snack habit		39.7 (25)	27.1 (16)	33.6 (41)	0.14
A family history of obesity		70.8 (46)	65.6 (40)	68.3 (86)	0.53
Co-morbidities					
Diabetes mellitus		6.5 (4)	0 (0)	3.3 (4)	**0.046**
Pulmonary diseases		1.6 (1)	0 (0)	0.8 (1)	0.32
Depression		1.6 (1)	8.3 (5)	4.9 (6)	4.9 (6)

*SD* Standard Deviation

Statistically significant results were shown in bold font

The anthropometric measurements of the individuals with obesity included in the study are presented in Table 2. Participants’ median BMI was 35.6 (30–56.4) kg/m^2^, mean weight was 98.9 ± 17.7 kg, median body fat percentage was 38.8% (24.1–47.1), mean total body fat mass was 38.2 ± 11.1 kg, mean total body muscle mass was 57.9 ± 9.9 kg, mean total fat-free mass was 61.1 ± 10.2 kg, total fluid mass was 44.1 ± 7.6 kg and total bone mass was 3.03 ± 0.5 kg. The distribution of body fat and muscle of the patients is shown in Table 2. The mean waist circumference was 105.9 ± 11.9 cm, median hip circumference was 124 (102–153) cm, and the mean waist/hip ratio was 0.85 ± 0.07 cm (Table 2).

**Table 2:** The comparison of anthropometric characteristics of individuals with obesity according to their metabolic phenotypes

	Metabolic phenotype			
	Metabolically unhealthy	Metabolically healthy	Total sample	
	Mean ± SD Median (min-max)	Mean ± SD Median (min-max)	Mean ± SD Median (min-max)	*p*
BMI (kg/m^2^)	36 (30.0–56.4)	35.5 (30.0–48.6)	35.6 (30.0–56.4)	0.39
Weight (kg)	100.3 ± 19.2	97.3 ± 16.09	98.9 ± 17.7	0.27
Body fat percentage (%)	37.9 (24.1–47.1)	39.2 (25.7–35.8)	38.8 (24.1–47.1)	0.5
Total body fat mass (kg)	38.7 ± 12.9	37.6 ± 8.8	38.2 ± 11.1	0.69
Total body muscle mass (kg)	59.1 ± 10.1	56.7 ± 9.7	57.9 ± 9.9	0.15
Fat-free mass (kg)	62.3 ± 10.4	59.9 ± 9.8	61.1 ± 10.2	0.13
Total body fluid mass (kg)	45.1 ± 8.1	43.1 ± 7.1	44.1 ± 7.6	0.14
Total bone mass (kg)	3.06 ± 0.5	3.01 ± 0.4	3.03 ± 0.5	0.27
Right leg fat mass (kg)	7.6 ± 2.5	7.7 ± 1.9	7.7 ± 2.2	0.78
Left leg fat mass (kg)	7.6 ± 2.4	7.6 ± 1.8	7.6 ± 2.1	0.65
Right arm fat mass (kg)	2.6 ± 1.06	2.6 ± 0.8	2.6 ± 0.9	0.79
Left arm fat mass (kg)	2.9 ± 1.2	2.8 ± 1.0	2.8 ± 1.1	0.96
Abdominal fat mass (kg)	15.8 (5.8–46.8)	16.6 (6.5–30.2)	16.4 (5.8–46.8)	0.40
Waist circumference (cm)	104.0 ± 12.4	105.7 ± 11.6	105.9 ± 11.9	0.94
Hip circumference (cm)	124.0 (102.0–153.0)	125.0 (104.0–147.0)	124 (102–153)	0.54
Waist/hip ratio	0.85 ± 0.07	0.84 ± 0.07	0.85 ± 0.07	0.22

*SD* Standard Deviation

Metabolically healthy and unhealthy obese groups were similar in terms of age and gender (*p* = 0.44; *p* = 0.39). There was no difference between the two groups in terms of the presence of childhood obesity, physical activity status, eating habits, and family history of obesity. Among co-morbidities, the frequency of having diabetes mellitus was statistically higher in the metabolically unhealthy group (*p* = 0.046) (Table 1).

There was no difference between the two groups in terms of BMI, weight, total body fat, muscle, fat-free, fluid, bone mass, distribution of body fat and muscle, waist, and hip circumference, and waist-hip ratio (Table 2).

The laboratory measurements of the individuals included in the study are shown in Table 3. Participants’ median fasting glucose level was 92.0 (61.0–144.0), median HbA1c level was 5.7 (4.5–8.0), median c-peptide level was 2.9 (1.2–7.6), median insulin level was 16.1 (2.7–93.4), median HOMA-IR was 3.7 (0.6–33.2), mean HDL and LDL levels were 49.4 ± 12.4 and 106.4 ± 29.5, median TG level was 118.5 (53.0–608.0).

**Table 3:** The comparison of laboratory findings of individuals with obesity according to their metabolic phenotypes

	Metabolic phenotype			
	Metabolically unhealthy	Metabolically healthy	Total sample	
	Mean ± SD Median (min-max)	Mean ± SD Median (min-max)	Mean ± SD Median (min-max)	*p*
Fasting blood glucose (mg/dl)	95.5 (71.0–144.0)	91.0 (61.0–99.0)	92.0 (61.0–144.0)	**0.016**
HbA1c (%)	6.0 (4.7–8.0)	5.4 (4.5–6.1)	5.7 (4.5–8.0)	**<0.001**
c-peptide (ng/ml)	3.2 (1.9–7.6)	2.6 (1.2–6.6)	2.9 (1.2–7.6)	**0.01**
Insulin	16.7 (2.7–93.4)	14.8 (2.8–48.7)	16.1 (2.7–93.4)	0.35
HOMA-IR	3.9 (0.6–33.2)	3.38 (0.6–33.2)	3.7 (0.6–33.2)	0.29
Total cholesterol (mg/dl)	185.0 (11.3–254.0)	173.0 (99.0–248.0)	177.0 (11.3–254.0)	0.06
HDL (mg/dl)	42.6 ± 9.2	56.6 ± 11.2	49.4 ± 12.4	**<0.001**
LDL (mg/dl)	114.2 ± 27.07	98.3 ± 29.9	106.4 ± 29.5	**0.003**
TG (mg/dl)	146.0 (53.0–608.0)	98.0 (5.0–155.0)	118.5 (53.0–608.0)	**<0.001**
Vitamin B12 (pg/ml)	294.0 (170.0–851.0)	324.5 (157.0–694.0)	320.0 (157–851.0)	0.49
Urea (mg/dl)	23.7 ± 6.8	23.07 ± 5.5	23.4 ± 6.2	0.64
Creatinine (mg)	0.8 ± 0.9	0.6 ± 0.1	0.7 ± 0.6	0.10
Uric acid (mg/dl)	5.05 ± 1.09	4.7 ± 1.1	4.9 ± 1.1	0.11
TSH (mIU/L)	2.08 ± 1.03	2.4 ± 1.4	2.2 ± 1.2	0.25
Vitamin D (IU)	13.0 (3.0–43.0)	13.6 (4.9–30.0)	13.0 (3.0–43.0)	0.63
İrisin (conc.pg/ml)	144.0 (10.1–3364.0)	158.1 (3.3–2666.0)	154.0 (3.3–3364.0)	0.73
NRG-4 (ng/ml)	0.7 (0.09–10.2)	0.7 (0.04–4.5)	0.7 (0.04–10.2)	0.39
FGF-21 (conc.pg/ml)	25.4 (0.4–291.3)	38.9 (1.8–461.8)	27.2 (0.4–461.8)	0.32

*SD* Standard Deviation, *HOMA-IR* Homeostatic Model Assessment for Insulin Resistance, *HDL* High Density Lipoprotein, *LDL* Low Density Lipoprotein, *TG* Triglyceride, *TSH* Thyroid Stimulating Hormone, *NRG-4* Neuregulin-4, *FGF-21* Fibroblast Growth Factor

Statistically significant results were shown in bold font

The median irisin, NRG-4, and FGF-21 levels were 154.0 (3.3–3364.0) conc.pg/ml, 0.7 (0.04–10.2) ng/ml ve 27.2 (0.4–461.8) conc.pg/ml, respectively (Table 3).

A comparison of laboratory values of obese participants according to their metabolic phenotypes is shown in Table 3. Fasting blood glucose, HbA1c, c-peptide, LDL and TG levels were statistically significantly higher in individuals with metabolically unhealthy obesity (*p* = 0.016; *p* < 0.001; *p* = 0.01; *p* = 0.03; *p* < 0.001), while HDL levels were statistically significantly higher in individuals with metabolically healthy obesity (*p* < 0.001) (Table 3).

No statistically significant difference was found between irisin, NRG4, and FGF21 levels between metabolically healthy and unhealthy individuals with obesity (Table 3).

It was found that irisin had a significant inverse relationship with BMI, right and left leg mass, right and left arm fat mass (r = −0.183, *p* = 0.044; r = −0.193, *p* = 0.033; r = −0.227, *p* = 0.012; r = −0.236, *p* = 0.009; r = −0.227, *p* = 0.012). NRG4 and FGF21 were not significantly associated with any anthropometric measurement of obese individuals (Table 4).

**Table 4:** The correlation of anthropometric measurements with brown fat-related hormones of the participants

		İrisin (conc.pg/ml)	NRG4 (ng/ml)	FGF21 (conc.pg/ml)
Age	r	0.175	−0.037	0.113
	*p*	0.055	0.685	0.217
BMI (kg/m^2^)	r	**−0.183**	0.001	0.137
	*p*	**0.044**	0.991	0.134
Weight (kg)	r	−0.118	0.007	0.019
	*p*	0.194	0.937	0.837
Fat percentage (%)	r	**−0.264**	−0.056	0.121
	*p*	**0.003**	0.541	0.186
Total body fat mass (kg)	r	−0.156	−0.037	0.104
	*p*	0.086	0.687	0.254
Total body muscle mass (kg)	r	0.016	0.050	−0.047
	*p*	0.857	0.587	0.610
Total body fluid mass (kg)	r	−0.006	0.011	−0.061
	*p*	0.946	0.903	0.504
Fat-free mass (kg)	r	0.007	0.040	−0.054
	*p*	0.936	0.663	0.557
Total bone mass (kg)	r	0.029	0.066	−0.035
	*p*	0.752	0.471	0.699
Right leg fat mass (kg)	r	**−0.193**	0.032	0.106
	*p*	**0.033**	0.728	0.243
Left leg fat mass (kg)	r	**−0.227**	0.014	0.144
	*p*	**0.012**	0.879	0.112
Right arm fat mass (kg)	r	**−0.236**	0.022	0.144
	*p*	**0.009**	0.811	0.113
Left arm fat mass (kg)	r	**−0.227**	0.015	0.140
	*p*	**0.012**	0.868	0.123
Abdominal fat mass (kg)	r	−0.139	0.003	−0.017
	*p*	0.127	0.973	0.851
Waist circumference (cm)	r	−0.078	0.028	0.144
	*p*	0.394	0.757	0.113
Hip circumference (cm)	r	−0.151	−0.056	0.087
	*p*	0.096	0.537	0.341
Waist/hip ratio	r	0.030	0.096	0.125
	*p*	0.746	0.294	0.170

*BMI* Body Mass Index

Statistically significant results were shown in bold font

When the correlation of laboratory measurements of obese individuals with brown adipose tissue-related hormones was analyzed, it was shown that irisin and vitamin D had a weak positive significant correlation (r = 0.219, *p* = 0.033), NRG4, and TG had a weak positive significant correlation (r = 0.209, *p* = 0.021). FGF21 was not significantly associated with any laboratory measurements (Table 5).

**Table 5:** The correlation of laboratory findings with brown fat-related hormones of participants

		İrisin (conc.pg/ml)	NRG4 (ng/ml)	FGF21 (conc.pg/ml)
Fasting blood glucose (mg/dl)	r	−0.139	−0.025	−0.027
	*p*	0.133	0.119	0.771
HbA1c (%)	r	−0.015	−0.007	−0.023
	*p*	0.873	0.937	0.800
c-peptide (ng/ml)	r	−0.094	−0.084	−0.090
	*p*	0.337	0.394	0.363
Insulin	r	−0.057	−0.047	0.005
	*p*	0.543	0.617	0.958
Total cholesterol (mg/dl)	r	0.064	−0.034	−0.045
	*p*	0.486	0.710	0.620
HDL (mg/dl)	r	0.021	0.025	0.161
	*p*	0.821	0.786	0.760
LDL (mg/dl)	r	0.031	−0.029	−0.127
	*p*	0.739	0.750	0.166
TG (mg/dl)	r	−0.030	**0.209**	−0.140
	*p*	0.739	**0.021**	0.125
Vitamin B12 (pg/ml)	r	0.037	−0.129	−0.069
	*p*	0.702	0.186	0.481
Urea (mg/dl)	r	0.037	0.126	0.060
	*p*	0.696	0.179	0.523
Creatinine (mg)	r	−0.005	−0.059	−0.078
	*p*	0.963	0.549	0.433
Uric acide (mg/dl)	r	−0.002	0.050	−0.174
	*p*	0.986	0.614	0.078
TSH (mIU/L)	r	−0.077	0.134	−0.006
	*p*	0.427	0.166	0.954
Vitamin D (IU)	r	**0.219**	−0.187	−0.085
	*p*	**0.033**	0.071	0.417

*HDL* High Density Lipoprotein, *LDL* Low Density Lipoprotein, *TG* Triglyceride, *TSH* Thyroid Stimulating Hormone

Statistically significant results were shown in bold font

When the metabolically healthy and unhealthy participants were stratified by dividing into male and female subgroups and compared the levels of FGF21, irisin, and NRG4 within each subgroup, no significant difference was found between subgroups (Table 6).

**Table 6:** The comparison of irisin, NRG-4 and FGF21 according to gender subgroups

	Metabolic phenotype									Gender					
	Metabolic unhealthy			Metabolic healthy			Total sample			Male			Female		
	Male median (min-max)	Female median (min-max)	*p*	Male median (min-max)	Female median (min-max)	*p*	Male median (min-max)	Female median (min-max)	*p*	Metabolic healthy median (min-max)	Metabolic unhealthy median (min-max)	*p*	Metabolic healthy median (min-max)	Metabolic unhealthy median (min-max)	*p*
İrisin (conc.pg/ml)	236.8 (19.1–2048)	119.9 (10.1–3364)	0.36	143.7 (41.5–1653)	158.1(3.3–2666)	0.52	190.2 (19.1–2048)	152.3 (3.3–3364)	0.22	143.7 (41.5–1653)	236.8 (19.1–2048)	0.86	158.1 (3.3–2666)	119.9 (10.1–3364)	0.61
NRG-4 (ng/ml)	1.09 (0.3–4.2)	0.7 (0.09–10.2)	0.45	0.87 (0.2–1.89)	0.69 (0.04–4.5)	0.98	0.9 (0.2–4.2)	0.7 (0.04–10.2)	0.47	0.9 (0.2–1.8)	1.09 (0.3–4.2)	0.44	0.7 (0.04–4.5)	0.8 (0.09–10.2)	0.59
FGF-21 (conc.pg/ml)	25.4 (0.4–291.3)	25.4 (0.4–19.3)	0.94	43.8 (4.6–182.2)	26.5 (1.8–461.3)	0.95	32.2 (3.5–182.2)	25.6 (0.4–461.8)	0.93	43.8 (4.6–182.2	26.9 (3.5–110.6)	0.35	26.5 (1.8–461.8)	25.4 (0.4–291.3)	0.49

## Discussion

Our study revealed that irisin, FGF21 and NRG4 levels of two groups with metabolically healthy and unhealthy obesity, which were matched in terms of age, gender, BMI, fat, muscle, lean body mass, fat, and muscle distribution were similar to each other.

Irisin is a myokine that has been shown to increase UCP-1 in adipose tissue and to have a browning effect on white adipose tissue. Due to these effects, it was thought to have a positive impact on metabolic health and a protective effect against obesity, however, the research conducted on the relationship between irisin and obesity did not conclude with the expected results. While some studies showed a positive significant correlation between weight, BMI, and body fat mass [19–22], others reported opposite results [23]. On the other hand, completely different from these studies, some other authors did not find any significant relationship between irisin and BMI [24]. The different results in all these studies can be attributed to the different sample sizes and study methods of the studies (participants with different BMIs, metabolic characteristics and ages). Nevertheless, most of the studies have shown that irisin levels are higher in individuals with obesity and increase in direct proportion to BMI and body fat amount [19–22]. This has been explained by the compensatory increase in irisin to protect against obesity as the level of obesity increases and the secretion of more irisin with the development of irisin resistance over time [22, 25]. All of the individuals included in our study were obese and no comparison was made with the persons without obesity, however, when the correlation of irisin, BMI, and fat percentage was examined, an inverse relationship was found between irisin, BMI, and fat percentage. This result supports the hypothesis that irisin is a protective hormone against obesity.

Although it is yet uncertain, it is considered that irisin may have a protective effect against obesity-related metabolic diseases such as type 2 diabetes, non-alcoholic fatty disease [25]. Hee et al. reported that irisin levels of patients with metabolic syndrome were higher than those without metabolic syndrome [21]. On the other hand, Yan et al. reported that irisin levels of individuals with metabolic syndrome were lower than those without metabolic syndrome [26]. In a study, which was similarly designed to the present study, irisin levels were found to be higher in individuals with metabolically healthy obesity than those with metabolically unhealthy obesity [27]. In our study, although irisin level was higher in the participants with metabolically healthy obesity, this difference was not statistically significant. It could be possible to reach statistical significance by increasing the sample size. There is no consensus in the literature on the relationship between metabolic syndrome and irisin, this may be due to the different samples of the studies and the inclusion of participants with different characteristics.

NRG4 has also been shown to maintain metabolic health without altering body weight in both normal and high- fed mice. Chen et al. found a positive correlation between adiposity parameters and NRG4 levels [28]. In another study conducted on people with metabolic syndrome, a positive correlation was also found between BMI and NRG4 levels [29]. In other studies, no relationship was found between obesity and NRG4 level [29–31]. In our study, no significant relationship was found between BMI, fat mass, and fat percentage. These results can be explained in several ways. Firstly, the study sample and population differences could have led to different results in the study. Secondly, due to the complex role of NRG4 in metabolism, unpredictable confounding factors may have given rise to the results.

Different results were found in studies investigating the relationship between metabolic health and NRG4 in individuals with obesity. In some studies, significantly lower levels of NRG4 were found in obese individuals with metabolic syndrome compared to those without metabolic syndrome. In some studies, significantly lower levels of NRG 4 were found in obese individuals with metabolic syndrome compared to those without metabolic syndrome [31–33]. It has also been presented that individuals with obesity have healthier metabolic profiles [34]. NRG4 is a hormone secreted from brown adipose tissue that regulates energy metabolism in people with obesity and is thought to prevent the development of metabolic disorders. The mechanism of protecting from metabolic disorders is supposed to be through reducing hepatic lipogenesis, increasing nutrient oxidation, and promoting a healthier adipokine profile to restore metabolic health impaired by an unhealthy diet. NRG4 depletion may lead to increased insulin resistance in adipocytes due to autophagy degradation of GLUT4 vesicules and inflammation [35]. All of this information explains the higher levels of NRG4 in metabolically healthy people with obesity. In our study, NRG4 levels of metabolically healthy and unhealthy groups were statistically similar unlike most of the findings in the literature. It is difficult to interpret this result, however, it can be explained in several ways. Factors such as the number of participants included in and different sample collection methods used in the studies could affect the results. The definitions of metabolically healthy and unhealthy obesity which could alter the study results could be dissimilar. In the present study, the definition of metabolically healthy and unhealthy obesity was clearly defined.

FGF21 is a newly discovered adipokine released from many organs, including brown adipose tissue, which is thought to prevent metabolic complications associated with obesity by increasing glucose utilization and fat oxidation in the liver [9, 36]. In studies comparing individuals with and without obesity [37, 38], FGF21 levels were correlated within BMI and fat mass [39, 40]. As with irisin and NRG4, FGF21 levels were higher in individuals with obesity suggesting that this increase could be a compensatory response or a result of resistance to FGF21 [36]. In our study, no correlation was found between FGF21, BMI, and fat mass. Different characteristics of the samples, the complex role of FGF21 in metabolism and unpredictable confounding factors could be the underlying causes of this situation.

In a study comparing individuals with and without metabolic syndrome, higher FGF21 levels were observed in patients with metabolic syndrome [36, 41]. In another study, when adjusted for age, sex, and BMI, FGF21 levels were twofold higher in individuals with metabolically unhealthy than people with metabolically healthy obesity [42]. In our study, although FGF21 levels were higher in individuals with metabolically healthy obesity, no statistical difference was found between FGF21 levels of participants with metabolically healthy and unhealthy obesity. Unlike other studies, we interpreted this result as FGF21 may not be a marker of metabolically healthy or unhealthy obesity, however, more comprehensive studies with larger samples should be performed to support this hypothesis.

In the present study, no significant correlation of irisin and FGF21 with any laboratory measurement was detected. When the literature was reviewed, conflicting results were observed about the correlation between both FGF21 and irisin and fasting glucose, HbA1c, insulin, HOMA-IR, and lipid parameters. Some studies found a positive correlation [21, 43, 44], while others found negative [26, 45] or no correlation [46] between these parameters.

In a study investigating the relationship between metabolic syndrome and NRG4 in persons with obesity, no relationship was detected between NRG4 and TG [31]. On the other hand, in the present study, only a weak correlation was found between TG and NRG4 when the relationship between NRG4 and metabolic parameters was examined. Similarly, there are also studies that showed a positive correlation between TG and NRG4 [47, 48].

NRG4 has been shown to activate liver ErbB3/ErbB4 signaling, inhibiting lipogenesis in the liver through liver X receptor (LXR) and sterol regulatory element-binding protein 1c (SREBP1c), causing overexpression of adipose triglyceride lipase gene, inhibiting expression of peroxisome proliferator-activated receptor gamma (PPAR γ), and ultimately inhibiting lipid accumulation in the liver, while also protecting against [49]. Based on this information, a negative correlation between NRG4 and triglyceride (TG) levels would be expected. However, in our study, a positive correlation between NRG4 levels and TG levels was observed. This result could be interpreted as the development of NRG4 receptor resistance and disruption of the Nrg4/ErbB signaling pathway due to increased adipose tissue in obese individuals, as suggested in a previous study [50]. Since all individuals in our study were obese and a significant correlation was observed particularly in metabolically unhealthy obese individuals, this interpretation is also appropriate for our study.

In our study, no significant correlation was found between FGF21 and glucose, insulin, and lipid parameters. Our study group did not have any comorbidities and the glucose and lipid values mostly did not exceed reference ranges. The discrepancy between our study results and the literature can be attributed to this.

Research has shown that there are distinct gender disparities in metabolic syndrome, with women often exhibiting a higher prevalence and different risk factors compared to men [51–54]. Based on this information, we investigated whether there are differences between male and female in terms of FGF21, NRG4, and irisin. In our study, when participants were stratified into male and female subgroups, we observed no significant difference in the levels of FGF21, irisin, and NRG4 between these subgroups. This finding suggests that the expression of these biomarkers is not influenced by gender in our sample population.

One of the strengths of our study is the comparison of two groups that are similar in terms of age, gender, BMI, fat and muscle mass, and fat and muscle distribution. Apart from metabolic unhealthiness, there was no difference between the two groups. Another strength of our study was the well-defined concepts of metabolic health and unhealthiness. Those without any metabolic risk factors other than obesity were considered metabolically healthy obese.

The most important limitation of the study is the inability to measure brown adipose tissue using imaging methods.

In conclusion, irisin, FGF21, and NRG-4 share the common characteristic of promoting the browning of white adipose tissue. Therefore, they have been considered to have protective effects against obesity and positive effects on metabolic health, and various studies have been conducted to explore their potential as therapeutic agents. However, in the present study, no difference was found in irisin, FGF21, and NRG4 levels between metabolically healthy and unhealthy obese individuals. Based on this finding, it can be suggested that these hormones may not have an effect on metabolic health. However, the discrepancies among studies in the literature indicate that the relationship between these hormones and metabolic health is highly complex and influenced by various factors, such as genetic factors, exercise status, dietary patterns, and inflammation. The weak association between irisin and BMI and body fat percentage may suggest a potential link between irisin with metabolic health. Therefore, further studies with larger sample sizes are needed.
